# Evaluation of rodent-only toxicology for early clinical trials with novel cancer therapeutics

**DOI:** 10.1038/sj.bjc.6690761

**Published:** 1999-11

**Authors:** D R Newell, S S Burtles, B W Fox, D I Jodrell, T A Connors

**Affiliations:** Medical School, University of Newcastle, Newcastle, UK; Drug Development Office, Cancer Research Campaign, 10 Cambridge Terrace, Regent’s Park, London, NW1 4JL, UK; Deceased; ICRF Medical Oncology Unit, Western General Hospital, Edinburgh, UK; School of Pharmacy, University of London, London, UK

**Keywords:** phase I trials, preclinical toxicology, starting dose

## Abstract

Preclinical toxicology studies are performed prior to phase I trials with novel cancer therapeutics to identify a safe clinical starting dose and potential human toxicities. The primary aim of this study was to evaluate the ability of rodent-only toxicology studies to identify a safe phase I trial starting dose. In addition, the ability of murine studies to predict the quantitative and qualitative human toxicology of cancer therapeutics was studied. Data for 25 cancer drugs were collated for which the preclinical and clinical routes and schedules of administration were either the same (22/25), or closely matched. The maximum tolerated dose/dose lethal to 10% of mice (MTD/LD_10_) was identified for 24 drugs, and in patients the maximum administered dose (MAD) was associated with dose-limiting toxicity (DLT) in initial clinical trials with 20 compounds. In addition, for 13 agents, the toxicity of the drug at one-tenth the mouse MTD/LD_10_ was also investigated in rats, following repeated administration (20 doses). A phase I trial starting dose of one-tenth the mouse MTD/LD_10_ (mg m^–2^) was, or would have been, safe for all 25 compounds. With the exception of nausea and vomiting, which cannot be assessed in rodents, other common DLTs were accurately predicted by the murine studies (i.e. 7/7 haematological and 3/3 neurological DLTs). For two of the 13 drugs studied in rats, repeated administration of one-tenth the mouse MTD/LD_10_ was toxic, leading to a reduction in the phase I trial starting dose; however, one-tenth the mouse MTD/LD_10_ was subsequently tolerated in patients. For the 20 drugs where clinical DLT was reached, the median ratio of the human MAD to the mouse MTD/LD_10_ was 2.6 (range 0.2–16) and the median ratio of the clinical starting dose to the MAD was 35 (range 2.3–160). In contrast, in 13 subsequent phase I trials with 11 of the initial 25 drugs, the median ratio of the clinical starting dose to the MAD was 2.8 (range 1.6–56), emphasizing the value of early clinical data in rapidly defining the dose range for therapeutic studies. For all 25 drugs studied, rodent-only toxicology provided a safe and rapid means of identifying the phase I trial starting dose and predicting commonly encountered DLTs. This study has shown that the routine use of a non-rodent species in preclinical toxicology studies prior to initial clinical trials with cancer therapeutics is not necessary. © 1999 Cancer Research Campaign


*British Journal of Cancer* (1999) **81**(5), 760–768
© 1999 Cancer Research Campaign
Article no. bjoc.1999.0761

### Evaluation of rodent-only toxicology for early clinical trials with novel cancer therapeutics

**DR Newell[1], SS Burtles[2], BW Fox[3], DI Jodrell[4] and TA Connors[5]**

[1]Medical School, University of Newcastle, Newcastle, UK; [2]Drug Development Office, Cancer Research Campaign, 10 Cambridge Terrace, Regent's Park, London NW1 4JL, UK; [3]Deceased; [4]ICRF Medical Oncology Unit, Western General Hospital, Edinburgh, UK; [5]School of Pharmacy, University of London, London, UK

This paper is dedicated to the memory of and achievements of Brian Fox

**Summary** Preclinical toxicology studies are performed prior to phase I trials with novel cancer therapeutics to identify a safe clinical starting dose and potential human toxicities. The primary aim of this study was to evaluate the ability of rodent-only toxicology studies to identify a safe phase I trial starting dose. In addition, the ability of murine studies to predict the quantitative and qualitative human toxicology of cancer therapeutics was studied. Data for 25 cancer drugs were collated for which the preclinical and clinical routes and schedules of administration were either the same (22/25), or closely matched. The maximum tolerated dose/dose lethal to 10% of mice ($MTD/LD_{10}$) was identified for 24 drugs, and in patients the maximum administered dose (MAD) was associated with dose-limiting toxicity (DLT) in initial clinical trials with 20 compounds. In addition, for 13 agents, the toxicity of the drug at one-tenth the mouse $MTD/LD_{10}$ was also investigated in rats, following repeated administration (20 doses). A phase I trial starting dose of one-tenth the mouse $MTD/LD_{10}$ (mg $m^{-2}$) was, or would have been, safe for all 25 compounds. With the exception of nausea and vomiting, which cannot be assessed in rodents, other common DLTs were accurately predicted by the murine studies (i.e. 7/7 haematological and 3/3 neurological DLTs). For two of the 13 drugs studied in rats, repeated administration of one-tenth the mouse $MTD/LD_{10}$ was toxic, leading to a reduction in the phase I trial starting dose; however, one-tenth the mouse $MTD/LD_{10}$ was subsequently tolerated in patients. For the 20 drugs where clinical DLT was reached, the median ratio of the human MAD to the mouse $MTD/LD_{10}$ was 2.6 (range 0.2–16) and the median ratio of the clinical starting dose to the MAD was 35 (range 2.3–160). In contrast, in 13 subsequent phase I trials with 11 of the initial 25 drugs, the median ratio of the clinical starting dose to the MAD was 2.8 (range 1.6–56), emphasizing the value of early clinical data in rapidly defining the dose range for therapeutic studies. For all 25 drugs studied, rodent-only toxicology provided a safe and rapid means of identifying the phase I trial starting dose and predicting commonly encountered DLTs. This study has shown that the routine use of a non-rodent species in preclinical toxicology studies prior to initial clinical trials with cancer therapeutics is not necessary. © 1999 Cancer Research Campaign

**Keywords:** phase I trials; preclinical toxicology; starting dose

The Phase I/II Clinical Trials Committee of the Cancer Research Campaign (CRC) was established in 1980 with the remit of expediting the early clinical evaluation of novel cancer therapeutics. In order to meet this objective, resources and facilities for the synthesis and formulation of new agents were made available, and through the Committee a network of phase I and phase II clinical investigators was established. In addition, it was recognized that safe, yet rapid, preclinical toxicology protocols would also be required if compounds were to progress efficiently into clinical trials. In designing the preclinical toxicology protocols, note was taken of a number of retrospective reviews which indicated that one-tenth of the mouse $LD_{10}$ (the dose lethal to 10% of mice treated), when doses are expressed on the basis of surface area (i.e. mg $m^{-2}$), represents a safe phase I trial starting dose (Freireich et al, 1966; Homan, 1972; Goldsmith et al, 1975; Penta et al, 1979; Rozencweig et al, 1981). More recent experience has largely confirmed the safety of selecting starting doses for phase I trials on the basis of the mouse toxicology data (Grieshaber and Marsoni, 1986; Penta et al, 1992; Arbuck et al, 1996). In practise, however, in many countries a non-rodent species, usually the dog, is still routinely used in preclinical toxicology studies with cancer therapeutics.

The preclinical toxicology protocols developed by the CRC, in conjunction with the European Organisation for Research and Treatment of Cancer (EORTC), took into account the accumulating data on the reliability of the mouse as a predictor of safe phase I clinical trial starting doses, and deliberately restricted studies to rodent-only investigations (Joint Steering Committee of the EORTC and CRC, 1990). In brief, these protocols included determination of the $MTD/LD_{10}$ (maximum tolerated dose/dose lethal to 10% of treated animals) in mice following intraperitoneal (i.p.), intravenous (i.v.) and, where appropriate, oral (p.o.) administration. Haematology, histopathology and bone marrow cytology were performed for up to 28 days after a single dose of the agent at a dose close to the $MTD/LD_{10}$, and after repeated dosing, usually daily for 5 days every week for 4 weeks. Lastly, the haematology, histopathology and bone marrow cytology studies were repeated in rats treated daily for 5 days every week for 4 weeks with one-tenth of the mouse $LD_{10}$, doses again being expressed as mg $m^{-2}$. The latter experiment was performed to check the safety of the proposed phase I trial starting dose in a second species, in an analogous manner to the use of the dog (Grieshaber and Marsoni, 1986). The protocols required that studies should be performed

*Received 12 January 1999*
*Revised 24 March 1999*
*Accepted 31 March 1999*

*Correspondence to:* S Burtles

For the Cancer Research Campaign Phase I/II Clinical Trials Committee

**Table 1** Details of compounds investigated in initial phase I trials

| Compound | Alternative names | Compound properties | Compound class | References |
|---|---|---|---|---|
| BZQ | | Aziridinyl quinone alkylating agent | Alkylating agent | Betteridge et al (1990); a. |
| CB10-277 | | Methylating agent | Alkylating agent | Foster et al, 1993a; 1993b |
| Clomesone | | Chloroethylating agent | Alkylating agent | b. |
| MDMS | | Bifunctional alkylating agent | Alkylating agent | Smith et al, 1987 |
| Mitozolomide | Azolastone | Chloroethylating agent | Alkylating agent | Newlands et al, 1985 |
| Temozolomide | Temodal®, methazolastone | Methylating agent | Alkylating agent | Newlands et al, 1992 |
| JM 216 | | Platinum IV complex | Platinum complex | McKeage et al, 1995; 1997 |
| AG337 | Nolatrexed, Thymitaq® | TS Inhibitor | Antimetabolite | Rafi et al, 1995; 1998 |
| Didox | | RNR Inhibitor | Antimetabolite | Veale et al, 1998; Carmichael et al, 1990 |
| MZPES | | Lipophilic DHFR inhibitor | Antimetabolite | Stuart et al, 1989 |
| CI 941 | DUP941, losoxantrone, biantrazole | Topoisomerase II inhibitor | Topoisomerase inhibitor | Foster et al, 1992 |
| DACA | XR5000 | Topoisomerase I and II inhibitor | Topoisomerase inhibitor | c. |
| Etoposide phosphate | Etopophos | Etoposide prodrug | Topoisomerase inhibitor | Millward et al, 1995 |
| 1069-C85 | | Tubulin binder | Tubulin binder | Judson et al, 1997 |
| Amphethinile | | Tubulin binder | Tubulin binder | Smith et al, 1988 |
| RSU1069 | | Aziridinyl nitroimidazole | Radiopotentiator | Horwich et al, 1986 |
| Bryostatin 1 | | Protein kinase C modulator | Signal transduction modifier | Prendiville et al, 1993; Philip et al, 1993; Jayson et al, 1995 |
| C6G mustard | | Galactose-targeted alkylating agent | Targeted agent | d. |
| PK-1 | | Polymer-targeted anthracycline | Targeted agent | Vasey et al, 1999 |
| Elactocin | | Antitumour antibiotic | Miscellaneous | Newlands et al, 1996 |
| FAA | LM975, mitoflaxone | Cytokine and blood flow modulator | Miscellaneous | Kerr et al, 1987 |
| LM985 | | FAA prodrug | Miscellaneous | Kerr et al, 1986 |
| Penclomedine | | Unknown | Miscellaneous | Jodrell et al, 1998 |
| SDZ 62–434 | | DNA synthesis inhibitor | Miscellaneous | e. |
| Trimelamol | CB10-375 | Pre-activated methylmelamine | Miscellaneous | Judson et al, 1989 |

TS, Thymidylate synthase; RNR, ribonucleotide reductase; DHFR, dihydrofolate reductase.
Personal communications from: a. E Gilby; b. J Green and SM Crawford; c. N Bleehen, C Twelves, I Judson, B Baguley; d. J Smyth; e. N Bleehen.

according to standards of Good Laboratory Practice (GLP), and used only male animals unless the drug was intended for human use in females or if there were known sex differences, in which case the most sensitive sex was used.

Recently, in the light of extensive experience within the CRC and EORTC, these rodent-only protocols have been revised (Burtles et al, 1995). Specifically, the protocols now focus on the use of only clinically relevant schedules, doses (MTD and below) and routes of administration (i.v. or p.o.). The emphasis in these revised protocols is on compound-specific toxicology, in order to both increase the clinical relevance of the results obtained and reduce the number of animals required. In addition, studies in rats now repeat directly those in mice to address the need for studies in two species and which, if either, of the two rodent species is more predictive of the subsequent human experience.

As of January 1998, the CRC had taken 44 novel cancer therapeutics, in which a small molecule drug forms all or part of the therapy, into phase I trial, i.e. this figure excludes antibody-alone and gene-based therapeutics. Of these 44 therapies, three were multi-component, i.e. CMDA or ZD2767 CPG2-A5B7 antibody-directed enzyme prodrug therapies (ADEPT) and PSC833-etoposide treatment (multidrug resistance modulation); five were anti-endocrine agents (4-hydroxyandrostenedione, abiraterone acetate (CB7630), idoxifene, pyridoglutethimide, zindoxifene); and for seven agents the phase I trial starting dose was derived from prior human experience in non-CRC studies (amsalog, 4-hydroxyanisole, eicosapentaenoic acid, D-limonene) or canine data (AG2034, CT2584, phyllanthoside). Four of the remaining 29 therapies are still under phase I investigation (AMD473, DMXAA, PK2 and SPAG), leaving 25 agents for which comparisons of preclinical rodent toxicology and clinical phase I trial results can be performed (Table 1).

The primary aim of the current study was to assess retrospectively the safety of using rodent-only toxicology in selecting phase I trial starting doses. In addition, this study has allowed:

1. A comparison of the quantitative toxicity of novel agents in mice and humans; i.e. a comparison of the MTD/$LD_{10}$ in mice and the MTD or MAD (maximum administered dose) in patients.
2. An analysis of the qualitative toxicology of compounds in mice and in humans; specifically, the ability of murine studies to predict dose-limiting and other human toxicities.
3. An evaluation of the utility of results from early clinical trials in rapidly optimising schedules for phase II (therapeutic) evaluation.

### MATERIALS AND METHODS

The 25 drugs included in the current analysis are listed in Table 1. Table 1 also summarizes the properties of the compounds, the general class to which each compound belongs and references to the reports describing the phase I clinical trials. The clinical studies were all approved by, and performed under the auspices of, the CRC Phase I/II Clinical Trials Committee. In addition, each trial was approved by the relevant local ethics committee (institutional review board).

Preclinical toxicology studies were performed according to the protocols previously published (Joint Steering Committee of the EORTC and CRC, 1990; Burtles et al, 1995), and the full reports are held on file at the Drug Development Office, CRC, London,

© 1999 Cancer Research Campaign 

**Table 2** Quantitative preclinical murine toxicology of the compounds investigated

| Compound | Route | Sex | Schedule | GLP | MTD/$LD_{10}$ ($mg\ kg^{-1}\ d^{-1}$) | Mouse MTD/$LD_{10}$ ($mg\ m^{-2}\ d^{-1}$) | Phase I start dose ($mg\ m^{-2}\ d^{-1}$) | Phase I start dose: Mouse MTD/$LD_{10}$ ratio |
|---|---|---|---|---|---|---|---|---|
| BZQ | i.v. | M/F | single | N | 1.1 | 3.3 | 0.25 | 0.08 |
| CB10-277 | i.v. | M | single | Y | 265 | 795 | 80 | 0.10 |
| Clomesone | i.v. | M | single | Y | 97 | 291 | 4 | 0.01 |
| MDMS | i.v. | M | single | Y | 47 | 141 | 14 | 0.10 |
| Mitozolomide | i.v. | M/F | single | N | 64 | 192 | 8 | 0.04 |
| Temozolomide | i.v. | M | single | Y | > 140 | > 420 | 50 | < 0.12 |
| JM 216 | oral | F | single | N | 200 | 600 | 60 | 0.10 |
| AG337 | i.v. | M/F | single | Y | 272 | 816 | 75 | 0.09 |
| Didox | i.v. | M | single | Y | 791 | 2373 | 192 | 0.08 |
| MZPES | i.v. | M | single | Y | 18 | 54 | 5.4 | 0.10 |
| CI 941 | i.v. | M | single | Y | 20 | 60 | 5 | 0.08 |
| DACA | i.v. | M | d × 5 | Y | 30 × 5 | 90 × 5 | 9 × 3 | 0.10 |
| Etoposide phosphate | i.p. | M | d × 5 | Y | 10 × 5 | 30 × 5 | 25 × 5 | 0.83 |
| 1069-C85 | oral | M | single | Y | 47 | 141 | 2.8 | 0.02 |
| Amphethinile | i.v. | M | single | Y | 137 | 411 | 40 | 0.10 |
| RSU 1069 | i.v. | M | single | Y | 150 | 450 | 35 | 0.08 |
| Bryostatin | i.v. | M | single | Y | 0.037 | 0.11 | 0.005 | 0.05 |
| C6G mustard | i.p. | M | single | N | 15 | 45 | 5 | 0.11 |
| PK-1 | i.v. | M | single | Y | 45 | 135 | 20 | 0.15 |
| Elactocin | i.v. | M | single | Y | 3.6 | 11 | 0.1 | 0.01 |
| FAA | i.v. | M/F | single | Y | 343 | 1029 | 500 | 0.49 |
| LM985 | i.v. | M | single | Y | 31 | 93 | 10 | 0.11 |
| Penclomedine | i.v. | M | d × 5 | Y | 80 × 5 | 240 × 5 | 22.5 × 5 | 0.09 |
| SDZ 62-434 | i.v. | M | single | Y | 15 | 45 | 4.5 | 0.10 |
| Trimelamol | i.v. | M | single | Y | 206 | 618 | 25 | 0.04 |

GLP, good laboratory practice. Note: mg/kg doses in mice were converted to mg $m^{-2}$ doses using a conversion factor of 3.

UK. Experiments were conducted according to local animal welfare regulations and were covered by a UK Home Office project licence.

### RESULTS

### Preclinical toxicology studies

The quantitative preclinical murine toxicology data on the 25 compounds studied are given in Table 2. The route and schedule of administration were those used in initial clinical studies with only three exceptions: etoposide phosphate and C6G mustard – where the clinical route was i.v. and not i.p.; and DACA – where the clinical schedule was daily ×3 and not daily ×5. With the exception of four drugs (BZQ, mitozolomide, JM216, C6G mustard), the preclinical toxicology studies were performed according to GLP.

Table 2 also lists the murine MTD/$LD_{10}$ data, the phase I trial starting doses and the ratios of the phase I trial starting dose to the mouse MTD/$LD_{10}$. In one case, temozolomide, a murine MTD/$LD_{10}$, could not be defined because of the limited solubility of the drug in the i.v. formulation (20% v/v dimethyl sulphoxide (DMSO) in saline). In all other cases, the MTD/$LD_{10}$ was defined, with a very broad range of potencies being observed (0.037–791 mg $kg^{-1}$ $day^{-1}$). Excluding temozolomide, the median (range) ratio of the phase I trial starting dose to the mouse MTD/$LD_{10}$ was 0.1 (0.01–0.83), and for 17/24 drugs the starting dose was within the range 5–15% of the mouse MTD/$LD_{10}$. For the other seven drugs there was a greater than 50% deviation from one-tenth of the mouse MTD/$LD_{10}$ as the phase I starting dose. The reasons for these deviations were as follows: clomesone (starting dose: MTD/$LD_{10}$ ratio = 0.01), due to a transcriptional error in the clinical protocol; mitozolomide (0.04), because of the clinical toxicities of other chloroethylating agents; etoposide phosphate (0.83), due to prior experience with etoposide; 1069C85 (0.02), and elactocin (0.01), because of marked toxicity in rats following repeated administration at one-tenth of the mouse MTD/$LD_{10}$ (see below); FAA (0.49), following prior experience with LM985; and trimelamol (0.04), due to prior experience with methylmelamines.

The qualitative murine toxicology data for the 25 drugs studied are summarized in Table 3. Three general categories of toxicology were recorded: clinical, macroscopic tissue pathology/histopathology and haematology/chemical pathology. In the case of chemical pathology, studies were not performed with all the drugs investigated as the requirement for such tests has only recently been introduced (Burtles et al, 1995). Furthermore, in describing clinical effects, non-specific signs such as piloerection and hypokinesia have not been reported. In addition to listing the toxicities observed with each drug, Table 3 also indicates the dose level at which the toxicity was observed. Although in the majority of cases the effects were observed at or below the i.v./p.o./i.p. MTD/$LD_{10}$, toxicities were in some cases only observed at higher dose levels or following repeated administration.

For 13 compounds, a repeat-dose i.p. toxicity study was performed in rats with five daily doses of one-tenth the mouse MTD/$LD_{10}$ being given every week for 4 weeks, i.e. 20 doses in total. As previously described (Joint Steering Committee of the EORTC and CRC, 1990), these studies were performed to confirm the safety of the proposed phase I trial starting dose in a second species. The drugs studied in rats were CB10-277, clomesone, temozolomide, didox, MZPES, CI941, etoposide phosphate, 1069C85, amphethinile, elactocin, LM975, SDZ 62-434 and trimelamol. In the case of temozolomide, one-tenth of the MAD and not MTD was used, as the latter could not be defined in mice due to the limited solubility of the drug in the i.v./i.p. vehicle (20% v/v DMSO in saline). With two exceptions, the only effects seen in

 © 1999 Cancer Research Campaign

**Table 3** Qualitative preclinical murine toxicology of the compounds investigated

| Compound | Clinical toxicity | Macroscopic pathology – histopathology | Haematology – chemical pathology |
|---|---|---|---|
| BZQ | nr | B/D. Testes | nr |
| CB10-277 | B. Weight loss | B. GIT, BM, testes. C. thymus, spleen, LN | B. RBC, WBC, platelets |
| Clomesone | A. Weight loss | A. Kidney. B. BM, LN, spleen, testes, thymus. D. CNS | B. WBC, RBC |
| MDMS | B. Weight loss | B. Testes, BM, lung, liver. C. GIT, LN | B. WBC |
| Mitozolomide | B. Weight loss. D. Alopecia | B. Bone marrow. C. Testes. D. LN, spleen, thymus, pancreas, kidney | B. WBC, D. RBC, renal, hepatic |
| Temozolomide | | B. GIT, testes, liver, spleen, LN, thymus. D. BM, lung | B. RBC, WBC, platelets |
| JM 216 | A. Weight loss | A. GIT. D. Liver | A. WBC, RBC, platelets |
| AG337 | A. Phlebitis. D. Weight loss | D. Testes, renal | |
| Didox | D. Weight loss | C. Lungs, GIT, BM | |
| MZPES | C. Convulsions | C. GIT | B. RBC |
| CI 941 | A. Weight loss | A. Testes, thymus. B. Spleen, LN, GIT, BM. D. Liver | B. RBC, WBC |
| DACA | A. Muscular spasm/convulsions | A. Testes, thymus, BM, GIT | A. RBC, WBC, hepatic |
| Etoposide phosphate | C. Weight loss | B. Testes. C. BM, liver, GIT, thymus, LN, salivary gland | NR |
| 1069-C85 | C. Weight loss, diarrhoea | B. Testes, GIT. C. Spleen, liver, thymus, LN, BM, heart, brain, skin, liver, bladder. D. Eye | B. Platelets. C. RBC, WBC |
| Amphethinile | B. Unsteady gait. D. Weight loss | B. Testes. C. Spleen, thymus, LN, BM, GIT. D. Liver, hair follicles | B. RBC, WBC, platelets |
| RSU 1069 | A. Phlebitis | A. Lungs. C. Kidneys, LN, GIT | |
| Bryostatin | A. Phlebitis. C. Weight loss | A. Liver, spleen, LN, thymus, BM, brain, lung, kidney, GIT | A. RBC, WBC, platelets |
| C6G mustard | NR | A. Kidney | A. WBC |
| PK-1 | A. Weight loss. D. Hind limb paralysis | A. Thymus, testes, spleen, liver, BM, GIT, LN. D. Kidney, neurones | A. RBC, WBC, platelets, hepatic |
| Elactocin | A. Weight loss, phlebitis. D. Diarrhoea | B. Thymus. C. LN, spleen, skin, BM, GIT, testes | B. RBC, WBC, platelets |
| FAA | A. Convulsions. D. Weight loss | NR | NR |
| LM985 | A. Convulsions. B. Loss of consciousness | B. Liver. D. Spleen | |
| Penclomedine | A. Neurotoxicity. C. Weight loss | C. BM, GIT, renal | A. WBC |
| SDZ 62-434 | | B. Liver, testes, thymus | |
| Trimelamol | C. Convulsions. D. Weight loss | C. Heart, testes, liver. D. BM | D. RBC |

NR, not reported. Notes: Dose level, route and schedule at which toxicity was observed: A. at the i.v./i.p. MTD/$LD_{10}$ or below, B. at the i.p. MTD/$LD_{10}$ or below, C. only at greater than the i.v./p.o./i.p. MTD $LD_{10}$, D. only after multiple i.p. dosing (daily × 5 or daily × 5 for 4 weeks). Toxicities: BM – bone marrow, LN – lymph node, GIT – gastrointestinal tract, RBC – anaemia, WBC – leucopenia, platelets – thrombocytopenia.

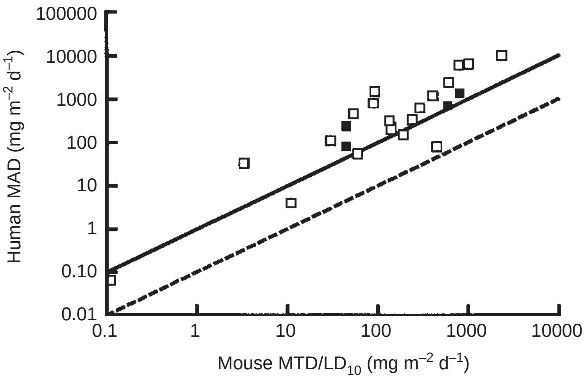

**Figure 1** Relationship between the mouse maximum tolerated dose/dose lethal to 10% of animals (MTD/$LD_{10}$) and the human maximum administered dose (MAD) for 24 anti-tumour agents. Each symbol is an individual drug; temozolomide is omitted as the mouse MTD/$LD_{10}$ was not defined. The open symbols are the drugs for which dose-limiting toxicity (DLT) was observed and the closed symbols are those where DLT was not observed in the initial clinical studies. The solid line is the line of identify (human MAD: mouse MTD/$LD_{10}$ = 1) and the broken line is the line for a phase I trial starting dose of one-tenth the mouse MTD/$LD_{10}$

rats following repeated dosing were minor reversible haematological changes (7/13), decreases in the rate of body weight gain (4/13) and testicular effects (2/13). However, for two compounds, 1069C85 and elactocin, there was significant toxicity following repeated administration to rats. With 1069C85 there was significant necrosis of the gastrointestinal tract and atrophy of lymphoid tissues at one-tenth the mouse MTD/$LD_{10}$, and with elactocin neither one-tenth nor one-hundreth of the mouse MTD/$LD_{10}$ were tolerated by rats on repeated dosing. On the basis of these rat data, the phase I trial starting doses of 1069C85 and elactocin were reduced to less than the one-tenth mouse MTD/$LD_{10}$ (Table 2).

### Initial phase I trial results

The quantitative details of the phase I trials initially performed with the 25 drugs are presented in Table 4. The median number of patients in the trials was 34, ranging from seven (RSU1069) to 64 (MZPES), and the median number of dose levels was eight, ranging from three (RSU1069) to 19 (clomesone). The dose escalation schema used in the phase I trials, which included arithmetic and 'modified Fibonacci' approaches, are given in the references cited in the Table 1. Table 4 also lists the human MAD, and for 20/25 drugs dose-limiting toxicity (DLT) was observed at or below the MAD. For the remaining five drugs, the initial clinical study was stopped before DLT was observed, either because administration was changed to an alternative route (temozolomide) or schedule (JM216, AG337), or because drug supplies were exhausted (C6G mustard and SDZ 62-434). Results of the subsequent clinical studies with temozolomide, JM216 and AG337 are discussed below.

Table 4 also compares the human MAD and the mouse MTD/$LD_{10}$, and gives the ratios of these two values. With the three exceptions indicated previously, i.e. DACA, etoposide phosphate and C6G mustard, the murine and clinical data are directly comparable in terms of route and schedule of administration. A comparison of the human MAD and the murine MTD/$LD_{10}$ values is shown graphically in Figure 1, and for the 20 drugs where DLT was observed at the human MAD, the median ratio of the human

**Table 4** Phase I trial details, maximum doses administered and ratios of human MAD to murine MTD/$LD_{10}$ doses

| Compound | Schedule | Starting dose (mg sq.m$^{-1}$ d$^{-1}$) | No. patients | No. dose levels | Human MAD (mg m$^{-2}$ d$^{-1}$) | Mouse MTD/$LD_{10}$ (mg m$^{-2}$ d$^{-}$) | Human MAD: Mouse MTD/$LD_{10}$ |
|---|---|---|---|---|---|---|---|
| BZQ | iv bolus | 0.25 | 34 | 15 | 33 | 3.3 | 10.0 |
| CB10-277 | Short i.v. infusion | 80 | 36 | 11 | 6000 | 795 | 7.5 |
| Clomesone | 30 min i.v. infusion | 4 | 63 | 19 | 639 | 291 | 2.2 |
| MDMS | iv bolus | 14 | 39 | 9 | 225 | 141 | 1.6 |
| Mitozolomide | 1-h i.v. infusion | 8 | 37 | 9 | 153 | 192 | 0.8 |
| Temozolomide | 1-h i.v. infusion | 50 | 16 | 4 | 200 | > 420 | > 0.48 |
| JM 216 | Single oral dose | 60 | 31 | 7 | 700 | 600 | 1.2 |
| AG337 | 24-h i.v. infusion | 75 | 13 | 6 | 1350 | 816 | 1.7 |
| Didox | Bolus – 30-min iv infusion | 192 | 34 | 14 | 10000 | 2373 | 4.2 |
| MZPES | 1-h iv infusion | 5.4 | 64 | 18 | 460 | 54 | 8.5 |
| CI 941 | iv bolus | 5 | 44 | 12 | 55 | 60 | 0.9 |
| DACA | 3-h i.v. infusion, d × 3 | 9 × 3 | 41 | 11 | 800 × 3 | 90 × 5 | 8.9 |
| Etoposide phosphate | 30–60 min i.v. infusion, d × 5 | 25 × 5 | 31 | 5 | 110 × 5 | 30 × 5 | 3.7 |
| 1069-C85 | Single oral dose | 2.8 | 39 | 8 | 200 | 141 | 1.4 |
| Amphethinile | Bolus – short i.v. infusion | 40 | 15 | 5 | 1200 | 411 | 2.9 |
| RSU 1069 | 15-min i.v. infusion | 35 | 7 | 3 | 80 | 450 | 0.2 |
| Bryostatin | 1-h i.v. infusion | 0.005 | 19 | 6 | 0.065 | 0.11 | 0.6 |
| C6G mustard | i.v. bolus | 5 | 35 | 8 | 80 | 45 | 1.8 |
| PK-1 | 1-h i.v. infusion | 20 | 36 | 8 | 320 | 135 | 2.4 |
| Elactocin | 1-h i.v. infusion | 0.1 | 10 | 6 | 4 | 11 | 0.4 |
| FAA | 1-h i.v. infusion | 500 | 27 | 8 | 6400 | 1029 | 6.2 |
| LM985 | 1-h i.v. infusion | 10 | 26 | 14 | 1500 | 93 | 16.1 |
| Penclomedine | 1-h i.v. infusion, d × 5 | 22.5 × 5 | 16 | 5 | 340 × 5 | 240 × 5 | 1.4 |
| SDZ 62-434 | i.v. bolus | 4.5 | 31 | 12 | 240 | 45 | 5.3 |
| Trimelamol | i.v. bolus | 25 | 49 | 14 | 2400 | 618 | 3.9 |

**Table 5** Qualitative human toxicology and predictive performance of preclinical murine studies

| Compound | Dose-limiting toxicity | Predicted | Other toxicities observed (not dose-limiting) | Predicted |
|---|---|---|---|---|
| BZQ | N&V | NE | Diarrhoea, alopecia, haematological | N, NR, NR |
| CB10-277 | N&V | NE | Flushing, diarrhoea, rash | NE, Y, NE |
| Clomesone | Haematological | Y | Cardiac, N&V, hepatic | N, NE, N |
| MDMS | Haematological | Y | N&V, alopecia, phlebitis | NE, N, N |
| Mitozolomide | Haematological | Y | N&V | NE |
| Temozolomide | Not reached | | Haematological | Y |
| JM 216 | Not reached | | N&V, diarrhoea, haematological, mucositis | NE, Y, Y, Y |
| AG337 | Not reached | | N&V, hepatic, phlebitis | NE, N, Y |
| Didox | Hepatic | N | Renal, N&V, hypotension, diarrhoea | N, NE, NE, Y |
| MZPES | N&V, neurotoxicity | NE, Y | Haematological | Y |
| CI 941 | Haematological | Y | N&V, mucositis, diarrhoea, alopecia, skin | NE, Y, Y, N, Y |
| DACA | Arm pain during infusion | NE | Flushing, N&V, haematological, chest pain | NE, NE, Y, NE |
| Etoposide phosphate | Haematological | Y | N&V, diarrhoea, hypersensitivity, mucositis, phlebitis, malaise, alopecia. | NE, Y, NE, Y, NE, NE, N |
| 1069-C85 | Neurotoxicity | Y | Haematological, diarrhoea, N&V, alopecia | Y, Y, NE, Y |
| Amphethinile | Not defined | | Neurotoxicity, N&V, alopecia, aesthenia, diarrhoea, haematological, pain | Y, NE, Y, NE, Y, Y, NE |
| RSU 1069 | N&V | NE | | |
| Bryostatin | Myalgia | NE | Lethargy, fever/rigors, hypotension, headache, haematological | NE, NE, NE, NE, Y |
| C6G mustard | Not reached | | N&V, mucositis | NE, N |
| PK-1 | Haematological, mucositis | Y, Y | N&V, alopecia, lethargy, hepatic, neurotoxicity | NE, N, NE, Y, Y |
| Elactocin | Aesthenia, N&V | NE, NE | Hepatic | N |
| FAA | Flushing | NE | N&V, myalgia | NE, NE |
| LM985 | Hypotension | NE | N&V, neurotoxicity | NE, Y |
| Penclomedine | Neurotoxicity | Y | N&V | NE |
| SDZ 62-434 | Not reached | | N&V, diarrhoea, headaches, neurotoxicity | NE, N, NE, N |
| Trimelamol | Haematological | Y | N&V, diarrhoea, aesthenia | NE, N, NE |

N&V, nausea and vomiting; NE, not evaluable in murine studies; NR, not reported in murine experiments.

MAD to the mouse MTD/$LD_{10}$ was 2.6, with a range of 0.2–16. For the same 20 drugs, the median ratio of the phase I trial starting dose to the eventual MAD was 35, ranging from 2.3 to 160, i.e. DLT was not observed at the starting dose for any these drugs. By definition, for the five drugs where DLT was not observed in the phase I trial, the phase I trial starting dose was also safe. Furthermore, for the five drugs (clomesone, mitozolomide, 1069C85, elactocin and trimelamol), where for various reasons (see above) the phase I trial starting doses were less than the one-tenth the mouse MTD/$LD_{10}$, DLT would not have been observed

 *© 1999 Cancer Research Campaign*

**Table 6** Details of subsequent phase I trials performed using results from initial studies (see Tables 4 and 5)

| Compound | Schedules | Starting dose mg $m^{-2}$ $d^{-1}$ | No. patients | No. dose levels | MAD mg $m^{-2}$ $d^{-1}$ | DLT |
|---|---|---|---|---|---|---|
| CB10-277 | 24-h i.v. infusion | 4700 | 22 | 5 | 15000 | Myelosuppression |
| Temozolomide | Oral, d × 1 | 200 | 35 | 12 | 1200 | Myelosuppression |
| Temozolomide | Oral, d × 5 | 150 × 5 | 42 | 4 | 240 × 5 | Myelosuppression |
| JM216 | Oral, d × 5 | 30 × 5 | 32 | 5 | 140 × 5 | Myelosuppression |
| AG337 | 120-h i.v. infusion | 96 × 5 | 32 | 9 | 1040 × 5 | Myelosuppression |
| Didox | 36-h i.v. infusion | 2500 × 3 | 12 | 4 | 7000 × 3 | Hepatic |
| MZPES | 24-h i.v. infusion | 460 | 6 | 3 | 800 | N&V, neurotoxicity |
| DACA | 3-h i.v. infusion | 18 | 32 | 9 | 1000 | Arm pain during infusion |
| Bryostatin | Various weekly | 0.025 | 35 | 4 | 0.05 | Myalgia |
| Bryostatin | 24-h i.v. infusion | 0.025 | 19 | 3 | 0.05 | Myalgia |
| Elactocin | Various | 1.5 | 23 | 5 | 4 × 5 | Aesthenia, N&V |
| FAA | 3- and 6-h i.v. infusion | 4800 | 27 | 5 | 10000 | Hypotension, diarrhoea |
| Trimelamol | i.v., d × 3 | 500 × 3 | 33 | 6 | 1000 × 3 | Myelosuppression |

N&V, nausea and vomiting.

even if one-tenth the mouse MTD/$LD_{10}$ had been used. This latter point is illustrated by the data points all being above the broken line in Figure 1.

The qualitative human toxicology data for the 25 compounds studied are presented in Table 5. Toxicities are distinguished as being either dose-limiting or not on the basis of the phase I trial reports (see Table 1). By comparing the data in Table 5 with those in Table 3, the ability of the preclinical murine studies to predict each human toxicity was determined. In comparing the human and murine data, it was recognized that a number of the human toxicities were either not evaluable or not evaluated in the preclinical experiments. The non-evaluable toxicities were nausea and vomiting, malaise/asthenia, flushing, fever/rigors, hypotension, headache, chest pain, hypersensitivity, rash, pain and myalgia. For the 20 drugs where DLT was observed, 22 toxic events were described as being dose-limiting. In the case of amphethinile, although DLT was reached, the exact nature of the DLT was not defined. The most common DLTs were haematological (7/22), nausea and vomiting (5/22) and neurotoxicity (3/22), the remaining DLTs only being reported on one occasion each. Of the human DLTs that were evaluated in the murine studies (12/22), 11 were correctly predicted, i.e. haematological seven, neurological three and mucositis one. Didox was the only drug where the human dose-limiting end organ, the liver, was studied in the preclinical experiments but toxicity was not observed. In total there were 78 other or non-DLTs reported, the most common being nausea and vomiting (18/78), diarrhoea (10/78), haematological (8/78), alopecia (7/78), malaise/asthenia (5/78), mucositis (4/78), hepatic (4/78) and neurological (4/78). Other toxicities occurred with an incidence of < 4 in 78 (i.e. < 5%). For the more common non-DLTs that were evaluated in the murine studies, the ability of the preclinical experiments to predict the human observations was as follows: diarrhoea 7/10, haematological 7/7, alopecia 2/6, mucositis 3/4, neurological 3/4 and hepatic 1/4.

### Subsequent phase I trial results

On the basis of the initial phase I trial results presented in Tables 4 and 5, 11 compounds were subject to a total of 13 further dose escalation studies in an attempt to optimize the dose, route and/or schedule of administration prior to therapeutic evaluation. As shown in Table 6, these additional studies involved a median of 32 patients per trial (range 6–42) and a median of 5 (range 3–12) dose levels. Importantly, the median ratio of the starting to the maximum dose administered was only 2.8 (range 1.6–56), i.e. a much smaller ratio than in the initial clinical trials (Table 4).

### DISCUSSION

The primary aim of the studies described in this paper was to evaluate the safety of initiating phase I trials with novel cancer therapeutics on the basis of rodent-only toxicology studies. Specifically, for all 24 drugs for which a mouse MTD/$LD_{10}$ was defined, one-tenth of this dose was, or would have been, safe in humans. With two compounds DLT was observed in patients at doses clearly less that the mouse MTD/$LD_{10}$, i.e. RSU1069 (DLT – nausea and vomiting) and elactocin (DLTs – asthenia/malaise and nausea/vomiting). However, for both drugs the DLTs were not evaluable in mice. Although for seven drugs the phase I trial starting dose was not in fact one-tenth the mouse MTD/$LD_{10}$, or close to it, had one-tenth the mouse MTD/$LD_{10}$ been used DLT would not have been encountered. Lastly, in the case of three compounds (AG337, flavone acetic acid and penclomidine) dog toxicology data were available at the time phase I trials were initiated; however, in all three cases the mouse was the most sensitive species and hence the phase I trial starting dose was based on the murine data.

The results reported here are in agreement with earlier retrospective reviews of the relationships between preclinical and clinical toxicology data (Freireich et al, 1966; Homan, 1972; Goldsmith et al, 1975; Penta et al, 1979; Rozencweig et al, 1981) as well as, in the main, more recent studies (Grieshaber and Marsoni, 1986; Penta et al, 1992; Arbuck et al, 1996). In these latter more recent reviews, which in some cases included certain of the drugs described here, a small number of drugs were identified where initiation of phase I trials at one-tenth of the mouse MTD/$LD_{10}$ would have exceeded the human MTD. The three most clear-cut instances were fludarabine (Grieshaber and Marsoni, 1986), tallimustine (Dent and Eisenhauer, 1996) and LY231514 (Dent and Eisenhauer, 1996). Both fludarabine and LY231514 are antimetabolites, and interspecies differences in the whole animal and cellular pharmacology of this class of drugs is well recognized. Of the 25 drugs

© 1999 Cancer Research Campaign studied by the CRC, three were antimetabolites (AG337, didox and MZPES) and for all three agents the murine toxicology safely identified a safe phase I trial starting dose. However, it should be noted that these three drugs are direct-acting antimetabolites, i.e. unlike classical antifolates and base/nucleoside analogues, they are not subject to intracellular metabolic activation. As such, the three antimetabolites studied here may not be representative of the drug class as a whole. Particular care needs to be taken in selecting phase I trial starting doses with antimetabolites, especially when the compound is known to undergo metabolic activation.

In general, the use of two species in preclinical toxicology studies is recommended in order to identify and compensate for marked interspecies differences. Most authorities require studies in one rodent and one non-rodent species, the non-rodent species usually being the dog. In reviewing data on 27 phase I trials with 17 new cytotoxic drugs, Dent and Eisenhauer (1996) concluded that dog toxicology data had appropriately influenced the choice of the phase I trial starting dose on three of the six occasions it was used (topotecan, LY231514, tallimustine). However, in reviewing data collated by Verweij (1996), Arbuck noted that the rat may also be able to safely identify a phase I trial starting dose, even when there were marked species differences in toxicology (Arbuck, 1996). In the current study, rat toxicology studies were only performed on a sub-set of 13 compounds, and then solely to check the safety of the proposed phase I trial starting dose, i.e. one-tenth the mouse $MTD/LD_{10}$, when given by repeated administration. Hence a comparison of the relative abilities of mouse and rat toxicology studies to predict quantitative and qualitative human toxicology data cannot be made on the basis of the results presented here. Recent modifications to the CRC/EORTC protocols will allow a direct comparison of mouse, rat and human toxicology data (Burtles et al, 1995), and results are currently being accumulated.

In addition to the primary aim of determining the safety of one-tenth of the mouse $MTD/LD_{10}$ as a phase I trial starting dose, this study has allowed a comparison of the quantitative and qualitative murine and human toxicologies for a range of drugs with widely varying structures, mechanisms of action and potencies. A comparison of the human MAD and the mouse $MTD/LD_{10}$, for drugs where clinical DLT was achieved, revealed a median ratio of 2.6 (range 0.2–16), a value and a range similar to those reported previously (Freireich et al, 1966; Homan, 1972; Goldsmith et al, 1975; Penta et al, 1979; Rozencweig et al, 1981; Grieshaber and Marsoni, 1986; Penta et al, 1992; Arbuck et al, 1996; Dent and Eisenhauer, 1996). With respect to the ability of the mouse to predict the qualitative nature of toxicities subsequently observed in humans, the data are again in agreement with earlier comparisons. Thus, haematological, neurological and antiproliferative-gastrointestinal human toxicities were predicted in most cases by the murine studies. Whilst it is recognized that mice, and to a lesser extent rats, do not allow investigations of the extent and sophistication possible in larger animals, the experience of the CRC is that this does not compromise patient safety, and that toxicities which more frequently become dose-limiting in human trials are detected in murine studies. In addition to systematically evaluating the rat in preclinical toxicology studies, recent revisions to the CRC/EORTC protocols (Burtles et al, 1995) include the routine use of chemical pathology studies, and these may help to identify less common side effects, e.g. renal, hepatic and cardiac toxicities, more reliably.

As noted by Dent and Eisenhauer (1996), phase I trials can be subdivided into those representing the first human experience with the drug, and those based on prior clinical data. In the current analysis, a similar distinction was made and the results obtained indicate that, once human data are available, subsequent clinical trials are in most cases conducted over a much smaller dose range, a median of 2.8 for the subsequent trials in Table 6 versus 35 for the initial trials listed in Table 4. Although clinical responses in phase I trials are rare (Estey et al, 1986; Decoster et al, 1990; Penta et al, 1992; Arbuck, 1996), they are more frequent at doses close to the MTD/recommended phase II dose (Penta et al, 1992), and hence it is important to minimize the number of patients treated in phase I trials at lower dose levels. From the data in Table 6, it is apparent that one approach to reducing the number of patients treated at doses that are unlikely to be effective is to rapidly obtain initial clinical data and then optimize the human dose, route and schedule of administration.

In the studies described in this paper, a range of approaches to dose escalation were used and, for the initial clinical studies (Table 4), the number of patients entered and dose levels required varied widely (median (range)), i.e. 34 (7–64) and 8 (3–19) respectively. Although it was not the aim of the current study to analyse the relative merits of the different dose escalation schemes used, the issues of dose escalation and phase I trial starting dose identification are intimately linked. Whilst the use of a 'homeopathic' phase I trial starting dose would invariably be safe, too conservative a starting dose can result in over lengthy dose escalation, time delays in starting therapeutic trials, the unnecessary use of clinical resources and large numbers of patients being treated at doses that are not even potentially therapeutic (Collins et al, 1986; Collins et al, 1990; Penta et al, 1992; Ratain et al, 1993; Simon et al, 1997). On the basis of the data presented here, one-tenth of the mouse $MTD/LD_{10}$ as a phase I trial starting dose appears to be a satisfactory compromise between a dose that is safe, but too low, and one that is more likely to be therapeutic, but also toxic. Once the phase I trial has been safely initiated, the challenge of rapidly identifying a dose for therapeutic evaluation should focus on the use of innovative dose escalation approaches including the application of all available preclinical data, pharmacologically guided dosing, model-based study designs and the use of pharmacodynamic trial-end points (Collins et al, 1986, 1990; EORTC Pharmacokinetics and Metabolism Group, 1987; O'Quigley and Chevret, 1991; Mick and Ratain, 1993; Ratain et al, 1993; Arbuck, 1996; Dent and Eisenhauer, 1996; Simon et al, 1997). In order to expedite such innovative designs it is essential that pharmacokinetic and pharmacodynamic investigations are included in preclinical toxicology studies wherever possible.

In considering the results of the preclinical and clinical studies described in this paper, and their implications for the identification of new cancer treatments, it is important to recognize the stages of clinical drug development these preclinical studies are intended to facilitate. The aim of the original and revised CRC/EORTC toxicology protocols (Joint Steering Group of the EORTC and CRC, 1990; Burtles et al, 1995) was to allow the rapid yet safe introduction of new cancer drugs into phase I trials and, provided acceptable clinical toxicology and pharmacology are observed, phase II therapy studies. The CRC/EORTC toxicology protocols are therefore intended to facilitate the equivalent of a United States Food and Drug Administration Investigational New Drug application (FDA-IND). In discussing regulatory considerations relevant to the preclinical development of anticancer drugs, DeGeorge and colleagues have recently emphasized the important distinction between studies required for an FDA-IND and those required to support a New Drug Application (NDA) (DeGeorge et al, 1998). In

 © 1999 Cancer Research Campaign the design of toxicology studies to support an NDA, these authors recognized the need to take into account the proposed therapeutic indication, the outcome of early clinical development, the nature of toxicities seen in animals and in humans, and the projected duration of clinical treatment. Whilst it is envisaged that the preclinical toxicology protocols used by the CRC/EORTC should ultimately facilitate NDA-type clinical trials, these protocols are not seen as a substitute for the more detailed compound-specific toxicology studies that may be required for product registration.

Although, of necessity, the study described in this paper constitutes a retrospective analysis, given the need to accrue both preclinical and clinical data, it represents the prospective evaluation of rodent-only preclinical toxicity studies. As such, the original hypothesis that rodent-only toxicity studies can be used to identify safe phase I trial starting doses has been tested and proven, for the 25 drugs studied. Of the 25 drugs investigated the majority (15) were conventional cytotoxic drugs (i.e. alkylating agents, antimetabolites, topoisomerase inhibitors or tubulin binding agents; Table 1), and hence caution must be exercised in extrapolating from the current results to newer classes of agents acting by novel mechanisms, e.g. mitogenic signal transduction inhibitors, anti-angiogenic and antimetastatic agents. However, clinical experience with these newer classes of drugs is currently insufficient to allow firm recommendations as to the most relevant preclinical toxicology models to use, and hence the emphasis should be on compound-specific protocols in the first instance. Specifically, in the context of the current study, it is not possible to comment on the relative merits of rodent and non-rodent species. In general, with the development of agents designed to exploit specific tumour-associated molecular lesions, the issue of target-related versus target-unrelated toxicity is likely to assume greater importance. In designing preclinical studies to address this issue, the increasing availability of gene knockout mice may have an important role to play. For example, toxicities seen in mice lacking the gene for the drug target must, by definition, be unrelated to the proposed mechanism of the anti-tumour action of the drug. Studies with gene knockout animals are likely to be restricted to rodents for the foreseeable future, and hence are complementary to the rodent-only approach described here.

In summary, the experience of the CRC in the phase I evaluation of 25 novel cancer therapeutics has shown that 1/10th the mouse $MTD/LD_{10}$ represented a safe Phase I trial starting dose for every drug. With the exception of nausea and vomiting, which cannot be evaluated in rodents, the more common human DLTs (haematological and neurological toxicity) were reliably predicted by the murine studies. These data do not support the routine use of a non-rodent species in preclinical toxicology studies prior to initial clinical trials with anticancer treatments.

### ACKNOWLEDGEMENTS

The authors wish to thank past and present members of the CRC Phase I/II Clinical Trials Committee for reviewing this manuscript, and for permission to cite unpublished and in press data. We are also grateful to the staff of the New Drug Development Office (Amsterdam, The Netherlands) and the EORTC Data Centre (Brussels, Belgium) for their input and helpful advice. The dedication and hard work of the staff of the CRC Drug Development Office is also greatly appreciated, without which this study would not have been possible. This work was supported by grants from the Cancer Research Campaign.

### REFERENCES

Arbuck SG (1996) Workshop on Phase I study design. *Ann Oncol* **7**: 567–573

Betteridge RF, Bosanquet AG and Gilby ED (1990) Pharmacokinetics of 2,5-diaziridinyl-3,6-bis(2-hydroxyethylamino)-1,4-benzoquinone (BZQ, NSC224070) during a Phase I clinical trial. *Eur J Cancer* **26**: 107–112

Burtles SS, Newell DR, Henrar REC and Connors TA (1995) Revisions of general guidelines for the preclinical toxicology of new cytotoxic anticancer agents in Europe. *Eur J Cancer* **31A**: 408–410

Carmichael J, Cantwell BMJ, Mannix KA, Veale D, Elford HL, Blackie R, Kerr DJ, Kaye SB and Harris AL (1990) A Phase I and pharmacokinetic study of didox administered by 36 hour infusion. *Br J Cancer* **61**: 447–450

Collins JM, Zaharko DS, Dedrick RL and Chabner BA (1986) Potential roles for preclinical pharmacology in Phase I clinical trials. *Cancer Treat Rep* **70**: 73–80

Collins JM, Grieshaber CK and Chabner BA (1990) Pharmacologically guided Phase I clinical trials based upon preclinical drug development. *J Natl Cancer Inst* **82**: 1321–1326

Decoster G, Stein G and Holdener EE (1990) Responses and toxic deaths in Phase I clinical trials. *Ann Oncol* **1**: 175–181

DeGeorge JJ, Ahn C-H, Andrews PA, Brower ME, Giorgio DW, Goheer ME, Lee-Ham DY, McGuinn WD, Schmidt W, Sun CJ and Tripathi SC (1998) Regulatory considerations for preclinical development of anticancer drugs. *Cancer Chemother Pharmacol* **41**: 173–185

Dent SF and Eisenhauer EA (1996) Phase I trial design: are new methodologies being put into practise? *Ann Oncol* **7**: 561–566

EORTC Pharmacokinetics and Metabolism Group (1987) Pharmacokinetically guided dose escalation in Phase I clinical trials. *Eur J Cancer* **23**: 1083–1087

Estey E, Hoth D, Simon R, Marsoni S, Leyland-Jones B and Wittes R (1986) Therapeutic response in Phase I trials of antineoplastic agents. *Cancer Treat Rep* **70**: 1105–1115

Foster BJ, Newell DR, Graham MA, Gumbrell LA, Jenns KE, Kaye SB and Calvert AH (1992) Phase I trial of the anthrapyrazole CI-941: prospective evaluation of pharmacologically guided dose-escalation. *Eur J Cancer* **28**: 463–469

Foster BJ, Newell DR, Carmichael J, Harris AL, Gumbrell LA, Jones M, Gogard PM and Calvert AH (1993*a*) Preclinical, Phase I and pharmacokinetic studies with the dimethyl phenyltriazene CB10-277. *Br J Cancer* **67**: 362–368.

Foster BJ, Newell DR, Gumbrell LA, Jenns KE and Calvert AH (1993*b*) Phase I trial with pharmacokinetics of CB10-277 given by 24 hours continuous infusion. *Br J Cancer* **67**: 369–373

Freireich EJ, Gehan EA, Rall DP, Schmidt LH and Skipper HE (1966) Quantitative comparison of toxicity of anticancer agents in mouse, rat, hamster, dog, monkey and man. *Cancer Chemother Rep* **50**: 219–244

Goldsmith MA, Slavik M and Carter SK (1975) Quantitative prediction of drug toxicity in humans from toxicology in small and large animals. *Cancer Res* **35**: 1354–1364

Grieshaber CK and Marsoni S (1986) Relationship of preclinical toxicology to findings in early clinical trials. *Cancer Treat Rep* **70**: 65–72

Homan ER (1972) Quantitative relationships between toxic doses of antitumour chemotherapeutic agents in animals and man. *Cancer Chemother Rep Part 3* **3**: 13–19

Horwich A, Holliday SB, Deacon JM and Peckham MJ (1986) A toxicity and pharmacokinetic study in man of the hypoxic-cell radiosensitizer RSU-1069. *Br J Radiol* **59**: 1238–1240

Jayson GC, Crowther D, Prendiville J, McGown AT, Scheid C, Stern P, Young R, Brenchley P, Chang J, Owens S and Pettit GR (1995) A Phase I trial of bryostatin 1 in patients with advanced malignancy using a 24 hour intravenous infusion. *Br J Cancer* **72**: 461–468

Jodrell DI, Bowman A, Stewart M, Dunlop N, French R, MacLellan A, Cummings J and Smyth JF (1998) Dose-limiting neurotoxicity in a Phase I study of penclomedine (NSC 388720, CRC 88-04), a synthetic α-picoline derivative, administered intravenously. *Br J Cancer* **77**: 808–811

Joint Steering Committee of the EORTC and CRC (1990) General guidelines for the preclinical toxicology of new cytotoxic anticancer agents in Europe. Eur J Cancer 26: 411–414

Judson IR, Calvert AH, Rutty CJ, Abel G, Gumbrell LA, Graham MA, Evans BD, Wilman DEV, Ashley SE and Cairnduff F (1989) Phase I trial and pharmacokinetics of trimelamol ($N^2$ $N^4$ $N^6$-trihydroxymethyl-$N^2$ $N^4$ $N^6$-trimethylmelamine). *Cancer Res* **49**: 5475–5479

Judson I, Briasoulis E, Raynaud F, Hanwell J, Berry C and Lacey H (1997) Phase I trial and pharmacokinetics of the tubulin inhibitor 1069C85, a synthetic agent binding at the colchicine site designed to overcome multidrug resistance. *Br J Cancer* **75**: 608–613

Kerr DJ, Kaye SB, Graham J, Cassidy J, Harding M, Setanoians A, McGrath JC, Vezin WR, Cunningham D, Forrest G and Soukop M (1986) Phase I and

© 1999 Cancer Research Campaign 

pharmacokinetic study of LM975 (flavone acetic acid ester). *Cancer Res* **46**: 3142–3146

Kerr DJ, Kaye SB, Cassidy J, Bradley C, Rankin EM, Adams L, Setanoians A, Young T, Forrest G, Soukop M and Clavel M (1987) Phase I and pharmacokinetic study of flavone acetic acid. *Cancer Res* **47**: 6776–6781

McKeage MJ, Mistry P, Ward J, Boxall FE, Loh S, O'Neill C, Ellis P, Kelland LR, Morgan SE, Murrer B, Santabarbara P, Harrap KR and Judson IR (1995) A Phase I and pharmacology study of an oral platinum complex, JM216: dose-dependent pharmacokinetics with single-dose administration. *Cancer Chemother Pharmacol* **36**: 451–458

McKeage MJ, Raynaud F, Ward J, Berry C, O'Dell D, Kelland LR, Murrer B, Santabarbara P, Harrap KR and Judson IR (1997) Phase I and pharmacokinetic study of an oral platinum complex given daily for 5 days in patients with cancer. *J Clin Oncol* **15**: 2691–2700

Mick R and Ratain MJ (1993) Model-guided determination of maximum tolerated dose in Phase I clinical trials: evidence for increased precision. *J Natl Cancer Inst* **85**: 217–223

Millward MJ, Newell DR, Mummaneni V, Igwemezie LN, Balmanno K, Charlton CJ, Gumbrell L, Lind MJ, Chapman F, Proctor M, Simmonds D, Cantwell BMJ, Taylor GA, McDaniel C, Winograd B, Kaul S, Barbaiya RH and Calvert AH (1995) Phase I and pharmacokinetic study of the water-soluble etoposide prodrug, etoposide phosphate (BMY-40481). *Eur J Cancer* **31A**: 2409–2411

Newlands ES, Blackledge G, Slack JA, Goddard C, Brindley CJ, Holden L and Stevens MFG (1985) Phase I clinical trial of mitozolomide. *Cancer Treat Rep* **69**: 801–805

Newlands ES, Blackledge GRP, Slack JA, Rustin GJS, Smith DB, Stuart NSA, Quarterman CP, Hoffman R, Stevens MFG, Brampton MH and Gibson AC (1992) Phase I trial of temozolomide (CCRG 81045: M&B 39831: NSC 362856). *Br J Cancer* **65**: 287–291

Newlands ES, Rustin GJS and Brampton MH (1996) Phase I trial of elactocin. *Br J Cancer* **74**: 648–649

O'Quigley J and Chevret S (1991) Methods for dose finding studies in cancer clinical trials: a review and results of a Monte Carlo study. *Stat Med* **10**: 1647–1664

Penta JS, Rozencweig M, Guarino AM and Muggia FM (1979) Mouse and large-animal toxicology studies of twelve antitumour agents: relevance to starting dose for Phase I clinical trials. *Cancer Chemother Pharmacol* **3**: 97–101

Penta JS, Rosner GL and Trump DL (1992) Choice of starting dose and escalation for Phase I studies of antitumour agents. *Cancer Chemother Pharmacol* **31**: 247–250

Phillip PA, Rea D, Thavasu P, Carmichael J, Stuart NSA, Rockett H, Talbot DC, Ganesan T, Pettit GR, Balkwill F and Harris AL (1993) Phase I study of bryostatin 1: assessment of interleukin 6 and tumour necrosis factor α induction in vivo. *J Natl Cancer Inst* **85**: 1812–1818

Prendiville J, Crowther D, Thatcher N, Woll PJ, Fox BW, McGown A, Testa N, Stern P, McDermott R, Potter M and Pettit GR (1993) A Phase I study of intravenous bryostatin 1 in patients with advanced cancer. *Br J Cancer* **68**: 418–424

Rafi I, Taylor GA, Calvete JA, Boddy AV, Balmanno K, Bailey N, Lind M, Calvert AH, Webber S, Jackson RJ, Johnston A, Clendeninn N and Newell DR (1995) Clinical pharmacokinetic and pharmacodynamic studies with the nonclassical antifolate thymidylate synthase inhibitor 3,4-dihydro-2-amino-6-methyl-4-oxo-5-(4-pyridylthio)-quinazoline dihydrochloride (AG337) given by 24-hour intravenous infusion. *Clin Cancer Res* **1**: 1275–1284

Rafi I, Boddy AV, Calvete JA, Taylor GA, Newell DR, Bailey NP, Lind MJ, Green M, Hine J, Johnston A, Clendeninn N and Calvert AH (1998) Preclinical and Phase I clinical studies with the non-classical antifolate thymidylate synthase inhibitor nolatrexed dihydrochloride given by prolonged administration in patients with solid tumours. *J Clin Oncol* **16**: 1131–1141

Ratain MJ, Mick R, Schilsky RL and Siegler M (1993) Statistical and ethical issues in the design and conduct of Phase I and Phase II clinical trials of new anticancer agents. *J Natl Cancer Inst* **85**: 1637–1643

Rozencweig M, Von Hoff DD, Staquet MJ, Schein PS, Penta JS, Goldin A, Muggia FM, Freireich EJ and DeVite VT (1981) Animal toxicology for early clinical trials with anticancer agents. *Cancer Clin Trials* **4**: 21–28

Simon R, Freidlin B, Rubinstein L, Arbuck S, Collins J and Christian MC (1997) Accelerated titration designs for Phase I clinical trials in oncology. *J Natl Cancer Inst* **89**: 1138–1147

Smith DB, Fox BW, Thatcher N, Steward WP, Scarffe JH, Wagstaff J, Vezin R and Crowther D (1987) Phase I clinical trial of methylene dimethyl sulfonate. *Cancer Treat Rep* **71**: 817–820

Smith DB, Ewen C, Mackintosh J, Fox BW, Thatcher N, Scarffe JH, Vezin R and Crowther D (1988) A Phase I trial and pharmacokinetic study of amphethinile. *Br J Cancer* **57**: 623–627

Stuart NSA, Crawford SM, Blackledge GRP, Newlands ES, Slack J, Hoffman R and Stevens MFG (1989) A Phase I study of meta-azidopyrimethamine ethanesulphonate (MZPES): a new dihydrofolate reductase inhibitor. *Cancer Chemother Pharmacol* **23**: 308–310

Vasey PA, Kaye SB, Morrison R, Twelves C, Wilson P, Duncan R, Thomson AH, Murray LS, Hilditch TE, Murray T, Burtles S, Fraier D, Frigerio E and Cassidy J (1999) *Clin Cancer Res* **5**: 83–94

Veale D, Carmichael J, Cantwell BMJ, Elford HL, Blackie R, Kerr DJ, Kaye SB and Harris AL (1988) A Phase I and pharmacokinetic study of didox: a ribonucleotide reductase inhibitor. *Br J Cancer* **58**: 70–72

Verweij J (1996) Starting dose levels for Phase I studies. *Ann Oncol* **7** (suppl. 1): 13

 *© 1999 Cancer Research Campaign*
